# Feasibility of structured endurance training and Mediterranean diet in *BRCA1* and *BRCA2* mutation carriers – an interventional randomized controlled multicenter trial (LIBRE-1)

**DOI:** 10.1186/s12885-017-3732-4

**Published:** 2017-11-10

**Authors:** Marion Kiechle, Ricarda Dukatz, Maryam Yahiaoui-Doktor, Anika Berling, Maryam Basrai, Vera Staiger, Uwe Niederberger, Nicole Marter, Jacqueline Lammert, Sabine Grill, Katharina Pfeifer, Kerstin Rhiem, Rita K. Schmutzler, Matthias Laudes, Michael Siniatchkin, Martin Halle, Stephan C. Bischoff, Christoph Engel

**Affiliations:** 1 Department of Gynecology and Center for Hereditary Breast and Ovarian Cancer, Klinikum rechts der Isar, Technical University Munich (TUM), Ismaninger Str. 22, 81675 Munich, Germany; 2 Institute for Medical Informatics, Statistics and Epidemiology, University of Leipzig, Haertelstrasse 16-18, 04107 Leipzig, Germany; 3 Department of Prevention and Sports Medicine, Klinikum rechts der Isar, Technical University Munich (TUM), Georg-Brauchle Ring 56, 80638 Munich, Germany; 4 Institute for Nutritional Medicine, University Hohenheim, Fruwirthstr. 12, 70593 Stuttgart, Germany; 5 Institute for Medical Psychology and Sociology, University Hospital Schleswig-Holstein, Campus Kiel, Preusserstr. 1 - 9, 24105 Kiel, Germany; 6 Center for Hereditary Breast and Ovarian Cancer, University Hospital Cologne, Kerpener Str. 34, 50931 Cologne, Germany; 7 Department of Internal Medicine 1, University Hospital Schleswig-Holstein, Campus Kiel, Arnold-Heller-Strasse 3, 24105 Kiel, Germany; 8 Else Kroener-Fresenius Prevention Center, Klinikum rechts der Isar, Technical University Munich (TUM), Ismaninger Str. 22, 81675 Munich, Germany

**Keywords:** BRCA1, BRCA2, Hereditary breast cancer, Hereditary ovarian cancer, Exercise, Mediterranean diet, Psychological support

## Abstract

**Background:**

Women with pathogenic *BRCA* germline mutations have an increased risk for breast and ovarian cancer that seems to be modified by life-style factors. Though, randomized trials investigating the impact of lifestyle interventions on cancer prevention and prognosis in *BRCA* carriers are still missing.

**Methods:**

We implemented a multicenter, prospective randomized controlled trial in *BRCA1/2* patients, comparing a lifestyle intervention group (IG) with a control group (CG) with the primary aim to prove feasibility. Intervention comprised a structured, individualized endurance training alongside nutrition education based on the Mediterranean diet (MD) for 3 months, plus monthly group training and regular telephone contact during the subsequent 9 months. The CG attended one session on healthy nutrition and the benefits of physical activity. Primary endpoints were feasibility, acceptance and satisfaction over 12 months. Furthermore, effects on physical fitness, diet profile, body mass index (BMI), quality of life and perceived stress were investigated.

**Results:**

Sixty-eight participants (mean age 41, mean BMI 23.2 kg/m^2^) were enrolled, of whom 55 (81%, 26 IG, 29 CG) completed 12 months. 73% (*n* = 26) participated in at least 70% of all intervention sessions. Predictors for drop-outs (19%; *n* = 13) or non-adherence (27%; *n* = 7) were not found. 73% rated the program highly and 80% would participate again. Severe adverse events did not occur. Positive effects in the IG compared to the CG were observed for secondary endpoints: BMI, MD eating pattern and stress levels.

**Conclusions:**

This lifestyle intervention was feasible, safe and well accepted. Positive results on eating habits, physical fitness and stress levels warrant a larger randomized trial.

**Trial registration:**

The study has been retrospectively registered at ClinicalTrials.gov (reference: NCT02087592) on March 12, 2014. The first patient was included on February 24, 2014.

## Background

Women with *BRCA* germline mutations have considerably increased lifetime risks for breast (55–60%) and ovarian (16–59%) cancer [1, 2]. However, current literature implies that the risk of developing cancer in gene carriers may be influenced through genetic factors (polymorphisms), as well as exogenous factors such as reproductive factors, lifestyle and physical activity during adolescence [3, 4]. The cancer risk is higher, if genotype carriers were obese, physically inactive during their youth, born after 1940 or had no children [5, 6]. Regular physical activity has a tremendous impact on breast cancer incidence, and mortality. The breast cancer risk of pre and postmenopausal women can be reduced by regular training on average by 25% [7]. Moreover, the engagement in regular physical training reduces the risks of recurrence and mortality in women with breast cancer by 50% [8] and leads to further advantages like gain in quality of life, increased fitness and improved tolerance of chemotherapy [9].

Hypercaloric nutrition leading to weight gain and obesity also increases the risk of breast cancer in both pre and postmenopausal women [10, 11]. Obese women with a BMI > 30 kg/m^2^ diseased with breast cancer have a greater risk of developing distant metastases and early mortality [12]. In a prospective study with sporadic breast cancer in patients under adjuvant standard therapy nutritional intervention with calorie and fat-restriction led to a significant reduction in recurrence rates [13]. Furthermore, a first randomized dietary intervention trial postulates a beneficial effect of the Mediterranean dietary (MD) pattern on breast cancer incidence [14]. This diet comprises a high intake of fruit and vegetables, whole grains, legumes, fish and olive oil, and low intakes of red meat and processed foods.

Furthermore, mental stress and depression play a significant role. Although not proven to play a significant role in primary prevention of breast cancer, an optimistic outlook on life and psychological well-being, health, and stress reduction have a positive effect in diseased breast cancer patients, accelerating recovery and even impairing mortality [15–17].

Although retrospective observations lead to the hypothesis that risk-modulating factors may also exist in *BRCA* associated cancers [5, 6], so far randomized trials investigating the impact of lifestyle interventions in prevention of cancer as well as on prognosis in *BRCA* carriers are missing.

Therefore, we aim to perform a prospective randomized intervention trial assessing, whether a long-term multifactorial lifestyle intervention program, including a structured physical endurance training and nutrition education stressing the MD pattern can lead to a reduction in breast cancer incidence and mortality in *BRCA1* and *BRCA2* mutation carriers [18]. Before initiation of this large trial, we have performed a pilot trial assessing feasibility and adherence in a smaller group which results we present here.

## Methods

### Study design

‘LIBRE-1’ (**L**ifestyle **I**ntervention study in women with hereditary **Bre**ast and ovarian cancer, 1 = pilot) [19] is a multicenter, prospective, two-armed randomized (1:1) controlled clinical trial including women with germline mutations in *BRCA1* or *BRCA2*. The primary aim of this study was to evaluate adherence to and acceptance of a structured, 1-year exercise program combined with a Mediterranean dietary pattern. A detailed description of the study design has been published [19]. The study has been registered at ClinicalTrials.gov (reference: NCT02087592). The ethics committee of all three participating university hospitals approved the study. The ethics review board of the faculty of medicine of the Technical University of Munich has approved the study protocol (Reference 5686/13) as well as the ethics committee of the faculty of Medicine of the University Cologne and Kiel (Reference 13-053 and Reference B 235/13).

Briefly, with written consent women aged 18–69 years with pathogenic germline mutations in *BRCA1* and *BRCA2* were recruited from three university hospitals in Germany (Cologne, Kiel and Munich), which are members of the German Consortium for Hereditary Breast and Ovarian Cancer (GC-HBOC), a national registry for *BRCA1/2* carriers. Exclusion criteria were medical problems not allowing exercise, e.g. metastatic tumor disease, cardiovascular and lung diseases or severe orthopedic problems; food allergies not allowing consumption of a Mediterranean diet; women who regularly exercise, pregnancy; BMI < 18 kg/m^2^ [19].

Sample size for this feasibility trial was determined pragmatically, using the recommended minimum of 30 participants per arm. The group allocation was done using a randomly permuted block randomization with an allocation ratio of 1:1. Randomization was stratified by center and previous diagnosis of cancer. While the CG received an introductory lecture on the positive effects of physical activity on the incidence and prognosis of breast cancer as well as a group lesson on healthy eating based on the recommendations of the German Nutrition Society (DGE), the IG received a structured lifestyle intervention program of increasing physical activity and over 12 months (3 months intensive phase followed by 9 months less intense supervision) [19]. Psychological support was not given, but was monitored by questionnaires (SSCS, EORTC QLQ-C30/−BR23, details see below) as were nutrition habits and level of physical activity. All parameters were assessed at baseline (SE/V0), after 3 (V1) and 12 months (V2). Data collection and measurements were performed in each study center and collected in a central electronic database (OpenClinica, Waltham, MA, USA) [19].

Outcomes to assess feasibility and acceptability of key trial parameters were participants’ completion of the study program and adherence to the intervention procedures. The primary endpoint of the study was the number of randomized women who successfully completed the first 3 months of the intervention program and remained a participant after 1 year. The study was considered feasible, if at least 70% of the participants completed 1 year of intervention. Additionally, recruitment and retention rates, safety and adverse events were assessed.

Secondary endpoints of the study included the measurements of quality of life (EORTC QLQ-C30−/BR23) [20, 21], perceived chronic stress (SSCS) [22], Body Mass Index (BMI), eating habits, nutrient and fat calorie intake (EPIC-FFQ) [23–25], adherence to the MD (MEDAS) [26], maximal oxygen intake (VO_2_ peak), ventilatory threshold (O_2_ at VT1) and physical activity (IPAQ) [27, 28].

### Statistical analysis

As this was a feasibility study, all statistical analyses were carried out with an exploratory and descriptive intention only. Thus, we deliberately did not adjust for multiple testing in this setting. All *p*-values are two-sided, and *p*-values < 0.05 were considered significant with hypothesis generating interpretation. Continuous variables were compared using non-parametric tests for independent (Kruskal-Wallis-Test, Mann-Whitney-U-Test) or paired groups (Friedman-Test, Wilcoxon-Test), where appropriate. Categorical variables were compared using the chi-square test. All statistical analyses were conducted using IBM SPSS Statistics for Windows Version 23.0 (IBM Corp, Armonk, NY, USA).

## Results

### Baseline characteristics

A total of 68 women with pathogenic *BRCA1* or *BRCA2* mutations were included in the study. 46 out of 68 (68%) participating females had been diagnosed with cancer before inclusion, 43 suffering from breast cancer, two had ovarian cancer and one with both ovarian and breast cancer.

Participants’ baseline data regarding health and *BRCA* mutation status as well as age in both the CG and IG are shown in Table 1. The groups did not differ regarding age, BMI or VO_2_peak. *BRCA* carriers were non-obese and of similar weight as the German average female population in this age group [29]. Median VO_2_max at baseline describing the cardio-pulmonary fitness status was 25.9 ml/kg/min in our cohort. This value was between Canadian breast cancer survivors (VO_2_peak of 21.4 ± 5 and O_2_ at VT1 of 14.9 ± 2.9 ml/kg/min) and healthy controls (29.1 ± 6 and 18.1 ± 3.5 ml/kg/min respectively) [30].

**Table 1:** Baseline characteristics of study participants

	Intervention group *n* = 33	Control group *n* = 35	Total *n* = 68	*P*-value
Cancer, n - Breast, n - Ovarian, n - both, n	23 21 1 1	23 22 1 0	46 43 2 1	0.728
*BRCA-1 / -2*, n	24 / 9	18 / 17	42 / 26	0.073
Age^a^, yrs	41 (27–72)	41 (24–68)	41 (24–72)	0.839
BMI^a^, kg/m^2^	22.2 (18.0–45.4)	23.6 (18.3–42.2)	23.2 (18.0–45.4)	0.482
VO_2_peak^a^, ml/kg/min	24 (12–42)	28 (15–38)	26 (12–42)	0.597
Drop-out, n	7	6	13	0.672

^a^Median (Range)

### Feasibility outcomes

#### Completion of the study

**Fig. 1:**
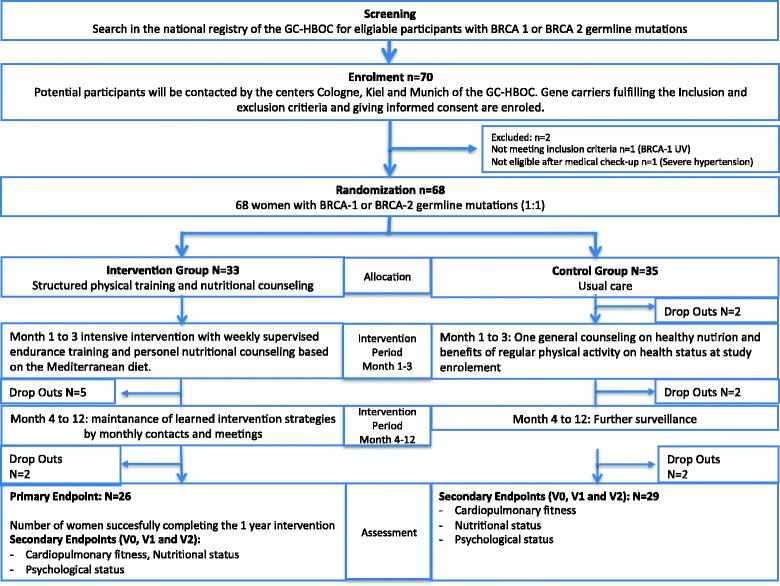
CONSORT flow diagram for LIBRE-1 pilot study [45]

Fifty-five out of 68 women completed the study after 1 year (81%) (Fig. 1). Of 33 participants allocated to the IG, 26 (79%) women completed 12 months. Five of seven participants discontinuing the intervention dropped out during the first 3 months (*n* = 4 because of low motivation, *n* = 3 disease-related). Drop-outs in the IG were slightly older and had a lower BMI compared to the non-drop-outs. There were no relevant differences regarding health status between the groups (Table 2).

**Table 2:** Baseline characteristic of drop-outs

Drop-outs	Intervention group *n* = 7	Control group *n* = 6	Total *n* = 13	*P*-value
Drop-out period, n				0.945
- Months 1–3 - Months 4–12	5 2	4 2	9 4	
Reason, n				0.445
- Motivation-related - Disease-related	4 3	5 1	9 4	
Cancer, n	5	4	9	0.945
Age^a^, years	45 (30–51)	34 (26–46)	39 (26–51)	0.138
BMI^a^, kg/m^2^	20.9 (19.8–26.9)	23.7 (20.7–38.6)	23.1 (19.8–38.6)	0.138
VO_2_peak^a^, ml/kg/min	30 (20–32)	24.5 (16–34)	26 (16–34)	0.543
O_2_ at VT1^a^, ml/kg/min	14 (9–19)	13.5 (10–25)	14 (9–25)	0.731
IPAQ, MET^a^min/wk	3366 (1552–12,561)	9750 (4278–14,085)	3990 (1552–14,085)	0.042

^a^Median (Range)

Six women in the CG discontinued the study, mainly for reasons related to lack of motivation (*n* = 5). Twenty-nine women (82%) of the CG completed the study program. Details of the drop-outs are shown in Table 2. In comparing baseline characteristics of drop-outs and non-drop-outs, no relevant differences in health or fitness status, activity levels, age or BMI were found. The results are shown in Table 2.

#### Adherence to intervention

The adherence to the intervention by participants who completed the 12 months of the study was relatively high and is illustrated in Table 3. Twenty-one of 26 women attended five or six of the six planned nutritional courses. The adherence to the training sessions was similar: 21 of the 26 women performed at least 26 of the 36 planned training sessions within the first 3 months. Combining both interventions, 19 out of 26 participants attended at least 70% of both the nutritional and physical training classes. The combined overall adherence rate was 73%. Participants with positive or negative adherence showed no significant differences regarding health or fitness status, activity level, age or BMI.

**Table 3:** Adherence to the lifestyle intervention program within the first 3 months and baseline characteristics (*n* = 26)

	Adherence positive (≥ 70% participation in the intervention sessions)	Adherence negative (< 70% participation in the intervention sessions)	*P*-value
Nutrition courses	21/26 (81%)	5/26 (19%)	
Cancer, n	16	2	0.610
Age*, years	43 (29–72)	41 (28–49)	0.686
BMI*, kg/m^2^	23 (18–45)	24 (20–37)	0.610
VO_2_peak*, ml/kg/min	24 (12–42)	23 (16–41)	0.952
O_2_ at VT1*, ml/kg/min	14 (8–28)	14 (10–21)	0.343
IPAQ, MET*min/wk	5130 (347–14,166)	6167 (4583–7812)	0.629
Training courses	21/26 (81%)	5/26 (19%)	
Cancer, n	14	4	0.114
Age*, years	42 (28–72)	41 (36–51)	0.286
BMI*, kg/m^2^	23 (18–45)	24 (20–37)	0.857
VO_2_peak*, ml/kg/min	24 (12–42)	22 (16–37)	0.467
O_2_ at VT1*, ml/kg/min	14 (8–28)	15 (10–21)	1.000
IPAQ, MET*min/wk	5376 (347–14,166)	5375 (2826–7812)	0.970
Nutrition and training	19/26 (73%)	7/26 (27%)	
Cancer, n	14	4	0.534
Age*, years	43 (29–27)	41 (28–51)	0.821
BMI*, kg/m^2^	23 (18–45)	24 (20–37)	0.778
VO_2_peak*, ml/kg/min	24 (12–42)	23 (16–41)	0.955
O_2_ at VT1*, ml/kg/min	14 (8–28)	15 (10–21)	0.642
IPAQ, MET*min/wk	5376 (347–14,166)	5576 (2826–7812)	0.974

*Median (Range)

#### Recruitment and retention

The first participant was recruited on the 24th of February 2014 and the last one on the 31st of July 2014. On the 15th of October 2015 the last participant finished the intervention program and completed V2. The 68 participants were recruited in less than 6 months. The recruitment rate per center was different: eight in Cologne, 23 in Kiel and 37 in Munich. Data entry was completed on April 30th, 2016. The retention period of the study (first patient in until data entry completion) was 26 months.

#### Adverse events and safety

Neither adverse events related to this study nor safety issues such as injuries during training were reported.

#### Satisfaction with the study

Eighteen out of 26 (69%) women of the IG who completed the study answered our questions on satisfaction with the study. 83% (15/18) attested grade 1 (good) or 2 (reasonable) regarding overall satisfaction with the study and care. 56% (10/18) judged the extent and feasibility of the physical training as good or reasonable. 78% (14/18) evaluated the nutritional intervention as good and feasible. 83% (15/18) would participate in such a study again.

Twenty two out of 29 (76%) participants of the CG, who completed the study answered the questionnaire. 64% (14/22) attested a good grade regarding overall satisfaction with the study and care. 41% (9/22) were satisfied with the general introductory lecture on positive effects of physical activity and healthy eating. 41% (9/22) evaluated the recommendations as good and feasible. 77% (17/22) would participate again in such a study. The results are presented in detail in Table 4.

**Table 4:** Participants satisfaction with the study

Rate of return	Intervention group 69% (18/26)			Control group 76% (22/29)			*P*-value
Items	“Good” Grading 1–2	“Inter-mediate” Grading 3–4	“Bad” Grading 5–6	“Good” Grading 1–2	“Inter-mediate” Grading 3–4	“Bad” Grading 5–6	
Overall satisfaction with the study and care	15 (83%)	3 (17%)	0 (0%)	14 (64%)	7 (32%)	1 (4.5%)	0.096
Extent and feasibility of physical intervention (IG)	10 (56%)	5 (28%)	3 (17%)	not asked			
Extent and feasibility of nutritional intervention (IG)	14 (78%)	4 (22%)	0 (0%)	not asked			
Extent and feasibility of general information on training and healthy eating (CG)	not asked			9 (41%)	10 (45%)	2 (9%)	
	yes	No		yes		no	*p*-value
Willingness to partake in such studies again	15 (83%)	3 (17%)		17 (77%)		5 (23%)	0.683

### Secondary outcome measurements

The secondary outcome measures are summarized in Table 5. The BMI was chosen as a secondary outcome measure representing physical exercise and nutrition. The baseline BMI values, as well as the measurements after 3 and 12 months (V1 and V2) showed no significant differences in the IG compared to the CG. However, by comparing the difference (Δ) of BMI from baseline to V1, there was a decrease in the IG compared to the CG (*p* = 0.002). This effect was not significant after 12 months (*p* = 0.115).

**Table 5:** Secondary endpoints

	Intervention group Median (range)	Control group Median (range)	*P*-values
Body Mass Index, kg/m^2^			
Baseline (V0)	22 .2 (18.0–45.2) (*n* = 33)	23.6 (18.3–42.7) (*n* = 35)	0.482
3 Months (V1)	23.4 (17.3–44.8) (*n* = 26)	24.4 (18.3–44.8) (*n* = 31)	0.804
12 Months (V2)	24.1 (18.6–46.3) *n* = 22)	23.3 (18.6–46.3) (*n* = 27)	0.833
Δ V1-V0	−0.19 (−4.7–0.8) (*n* = 26)	0.32 (−1.1–2.2) (*n* = 31)	0.002
Δ V2-V0	−0.16 (−7.6–2.8) (*n* = 22)	0.034 (−1.9–3.6) (*n* = 27)	0.115
EPIQ-FFQ: energy intake, kcal/day			
Baseline (V0)	1955.2 (863.8–3530.5) (*n* = 32)	2245.4 (1060.4–3088.3) (*n* = 32)	**0.045**
3 Months (V1)	2240.2 (800.7–3673.0) (*n* = 25)	2085.8 (780.7–2965.8) (*n* = 29)	0.761
12 Months (V2)	2024.6 (989.0–3516.9) (*n* = 25)	1936.0 (308.1–3609.9) (*n* = 27)	0.405
Δ V1-V0	78.8 (−1048.1–834.1) (*n* = 24)	−149.4 (−741.4–653.7) (*n* = 28)	0.119
Δ V2-V0	133.3 (−962.3–634.0) (*n* = 24)	−112.6 (−1383.2–547.8) (*n* = 26)	**0.007**
EPIQ-FFQ: Fat calorie intake [%energy intake]			
Baseline (V0)	40.1 (28.7–67.9) (*n* = 32)	40.4 (31.4–50.0) (*n* = 32)	0.968
3 Months (V1)	39.4 (31.3–63.1) (*n* = 25)	40.3 (30.9–60.0) (*n* = 29)	0.910
12 Months (V2)	40.2 (33.5–66.9) (*n* = 25)	39.2 (27.1–50.6) (*n* = 27)	0.504
Δ V1 - V0	0.35 (−14.32–8.38) (*n* = 24)	0.49 (−11.01–15.15) (*n* = 28)	0.971
Δ V2 - V0	−1.30 (−6.69–26.61) (*n* = 24)	−2.22 (−13.16–14.25) (*n* = 26)	0.367
MEDAS: (0–14 score points)			
Baseline (V0)	7 (2–10) (*n* = 33)	5 (3–11) (*n* = 31)	0.020
3 Months (V1)	9 (6–13) (*n* = 26)	6 (3–12) (*n* = 29)	0.001
12 Months (V2)	8 (5–13) (*n* = 25)	6 (2–13) (*n* = 27)	0.001
Δ V1-V0	2 (−1–8) (*n* = 26)	1 (−3–4) (*n* = 28)	0.110
Δ V2-V0	2 (−2–6) (*n* = 25)	0 (−3–6) (*n* = 25)	0.044
IPAQ, MET*min/wk			
Baseline (V0)	4583 (347–14,166) (*n* = 31)	4215 (300–15,624) (*n* = 29)	0.294
3 Months (V1)	4447 (834–11,904) (*n* = 26)	3230 (173–37,788) (*n* = 30)	0.212
12 Months (V2)	3754 (1012–11,706) (*n* = 24)	4528 (189–56,943) (*n* = 28)	0.463
Δ V1-V0	252 (−7961–4878) (*n* = 25)	−125 (−14,085–22,164) [27]	0.654
Δ V2-V0	−1878 (−10,236–6084) (*n* = 22)	961 (−4178–41,310) (*n* = 24)	0.004
VO_2_peak, ml/kg/min			
Baseline (SE-V0)	24 (12–42) (*n* = 33)	28 (15–38) (*n* = 35)	0.597
3 Months (V1)	26 (15–42) (*n* = 25)	27 (14–40) (*n* = 30)	0.993
12 Months (V2)	24 (10–35) (*n* = 22)	26 (14–44) (*n* = 23)	0.459
Δ V1-V0	2 (−6–10) (*n* = 25)	0 (−7–6) (*n* = 30)	0.146
Δ V2-V0	−1 (−13–11) (*n* = 22)	−3 (−9–6) (*n* = 23)	0.045
O_2_ at VT1, ml/kg/min			
Baseline (SE/V0)	14 (8–28) (*n* = 32)	16 (10–28) (*n* = 35)	0.281
3 Months (V1)	15 (9–28) (*n* = 23)	15 (10–39) (*n* = 30)	0.787
12 Months (V2)	14 (6–26) (*n* = 22)	16 (8–28) (*n* = 23)	0.068
Δ V1-V0	1 (−11–5) (*n* = 22)	−1.5 (−9–18) (*n* = 30)	0.019
Δ V2-V0	0 (−12–8) (*n* = 21)	0 (−10–7) (*n* = 23)	1.000
QLQ-C30 scale 1 (0–100 scores)			
Baseline (SE/V0)	68.7 (17–100) (*n* = 33)	69.1 (33–100) (*n* = 35)	0.938
3 Months (V1)	69.9 (0–100) (*n* = 26)	73.3 (17–100) (*n* = 30)	0.569
12 Months (V2)	70.1 (25–100) (*n* = 24)	63.1 (8–100) (*n* = 26)	0.309
Δ V1-V0	1.3 (*n* = 26)	3.3 (*n* = 30)	0.603
Δ V2-V0	2.1 (*n* = 24)	−4.8 (*n* = 26)	0.267
SSCS (0–48 scores)			
Baseline (SE/V0)	15.3 (3–38) (*n* = 33)	19.5 (0–38) (*n* = 35)	0.062
3 Months (V1)	16.0 (3–37) (*n* = 26)	18.2 (0–39) (*n* = 29)	0.339
12 Months (V2)	14.6 (3–41) (*n* = 22)	20.9 (1–39) (*n* = 27)	0.022
Δ V1-V0	0.4 (*n* = 26)	−0.9 (*n* = 29)	0.388
Δ V2-V0	−0.6 (*n* = 22)	1.44 (*n* = 27)	0.218

*Median (Range)

Bold = significant *P*-values

Regarding nutrition the total daily calorie intake (TEI) measured by a questionnaire (EPIQ-FFQ) showed no differences in the intervention and control group at baseline, after 3 (V1) and 12 (V2) months. The amount of calorie intake was in median 1965–2234 kcal per day. Women of the control group reduced their total calorie intake (approximately 250 kcal/day) after 12 months compared to the intervention group (*p* = 0.007). The amount of dietary fat accounts to about 39–40% of TEI with no difference between groups at any time point. A detailed subgroup analysis of macronutrient intake and micronutrient profiles will be published separately.

The median MEDAS being 7 (2–10) at baseline in the IG is two score points higher compared to a median of 5 (3–11) in the CG (*p* = 0.020). At V1 the difference is more considerable: 9 (6–13) versus 6 (3–12; *p* = 0.001), but again reveals a consistent group difference of two score points at V2 being 8 (5–13) in the IG and 6 (2–13) in the CG (*p* = 0.001). When the MEDAS delta between V2 and baseline is compared between groups, the increase in the IG appears to be relevant (*p* = 0.044).

Data on physical activity and physical fitness during intervention varied substantially among individuals and were not conclusive. VO_2_peak improved in the IG after 3 months, but these effects diminished after 12 months, a finding often seen in intervention trials, as contact decreases. Also aerobic capacity (O_2_ at VT1), an additional parameter of basic fitness did not improve (Table 5). These objectively measured data were not in line with subjective assessment of physical activity by the IPAQ questionnaire. While activity increased during intervention in the CG and even decreased in the IG, cardio-pulmonary parameters changed vice versa (Table 5).

The screening scale data of chronic stress (SSCS) in the study population showed similar scores compared to a reference cohort of the German population (age 31–59 years) [22]. The standard value for this age group is a score of 13 with a reference range of 6 to 24 score points [22]. The median SSCS scores of the IG and CG were always within the standard range, however revealed higher individual ranges from 0 to 41 scores compared to the reference range of 6 to 24 scores. This indicates that women with chronic stress experience have probably been included. After 12 months the participants of the IG significantly improved stress scores compared to the CG (IG: 14.6 ± 3–41; CG: 20.9 ± 1–39; *p* = 0.022).

The health related quality of life was measured by EORTC QLQ-C30. Scale 1 measuring global health status and quality of life showed no significant differences between IG and CG at any time points. The median scores (63.1–73.3) were within the reference range [31]. More detailed data of the additional 14 scales of the EORTC QLQ-C30 as well as the eight scales of breast cancer specific module EORTC QLQ-BR23 will be published separately.

## Discussion

LIBRE-1 is worldwide the first prospective randomized multicenter lifestyle intervention trial in *BRCA1/2* mutation carriers. This pilot study demonstrated the feasibility of recruiting and retaining women in a demanding and time-consuming structured intervention program including regular exercise (three exercise classes per week for 3 months) and nutrition education stressing the Mediterranean diet (six courses within 3 months). Additionally, we observed a favorable effect on BMI, chronic stress levels and changes in nutritional habits towards a Mediterranean eating pattern.

First and foremost, the intervention appeared safe and was not associated with any adverse events. Furthermore, recruitment of the planned number of participants (*n* = 60) was relatively fast, in less than 6 months 68 women with *BRCA* germline mutations were enrolled. The *BRCA* carriers were not obese and their BMI comparable to the healthy general population [29]. Median baseline fitness levels seemed to be below average, however, the group investigated was small and 68% were diseased. The median VO_2_peak was comparable to German breast cancer patients after chemotherapy [32, 33].

Motivation and adherence to the intervention was high. This might be explained by awareness of increased lifetime risks of cancer (55–60% for breast and 16–59% for ovarian cancer) [2] as well as the fact of being diagnosed with cancer before. This resulted in an overall completion rate of 81% after 12 months. 21% of the women allocated to the intervention and 18% randomized to the control group discontinued the study. In the control group the main reason for abandoning the study was the disappointment of not being randomized to the intervention group. In the intervention group the reasons were mainly lack of motivation, but also disease-related like progression of cancer or new diagnosis of cancer. However, the high overall acceptance rate and relatively low rate of attrition suggest that the intervention was well received. Moreover, age, BMI, health or fitness status had no influence on drop-out rates.

In comparable randomized pilot lifestyle intervention trials in breast cancer patients, similar but also higher drop-out rates have been reported ranging from 19 to 41% in exercise intervention studies [34] and in nutritional intervention between 17 and 23% [13, 35]. In a randomized feasibility study similar to ours combining nutritional and training intervention for 3 months performed within the Scottish breast cancer-screening program, the same total drop-out rate of 19% as in our study was found [36]. Interestingly, in the Scottish study cohort 44% of the 80 participants reported a positive family history of breast cancer. This underlines the hypothesis that breast cancer awareness itself resulting e.g. from a positive family history will improve attitude towards adapting lifestyle habits.

Adherence to the intervention program (> 70% of the intervention classes (18)) was an additional important parameter for assessing feasibility of the LIBRE trial. Combining results of nutrition and exercise training courses, 73% of the women in the intervention group fulfilled the 70% threshold. 81% of the participants in the intervention group achieved the exercise training requirements. Compared to other lifestyle intervention trials these adherence rates were high [37]. In previous trials adherence to dietary regimens was higher than for exercise intervention [38]. Therefore, we conclude that our intervention program is feasible and practicable.

Moreover, the participants attested a high satisfaction with the study and care within the trial, especially those allocated to the intervention arm. Only 17% stated that the physical training was not feasible or too extensive. Regarding nutritional intervention no concerns were reported. The rating of the control group was slightly worse compared to the intervention group regarding overall satisfaction with the study and care, re-participation, extent and feasibility of the lifestyle intervention. This may be explained by the women’s disappointment of having been randomized to the control group. This is supported by the fact that predictors for adherence or non-adherence could not be identified. Age, BMI, health or fitness status, which may have influenced adherence, were not different between the groups. However, numbers of women enrolled are still rather small and we will further investigate this issue in the subsequent larger LIBRE-2 trial.

We observed some significant effects on secondary outcomes in the intervention group compared to the controls (Table 5). However, since multiple comparisons were performed between study arms, these significant findings have to be interpreted with great caution. Due to the exploratory nature of this pilot study, we deliberately did not correct for multiple testing. Thus, significant findings can only be interpreted as hypothesis generating rather than confirmatory results. Moreover, because of the low sample size of this pilot study the statistical power to detect truly existing differences between the study arms with regard to secondary outcome measures was low.

The participants’ calorie intake corresponded to the reference values of energy requirements for medium physical activity, according to recommendations of the German (D), Austrian (A) and Swiss (CH) Nutrition Societies (D-A-CH reference values). These adopt recommendations of the EFSA - Scientific Opinion on Dietary Reference Values for energy [39]. However, the calorie intake of our study population was somewhat above the median intake of 1833 kcal/day, as described in the German National Nutrition Survey II (NVS II), the study forming the basis of the analysis of Heuer et al. [40]. Interestingly, caloric intake in the intervention group did in fact slightly increase over 3 or 12 months, but was slightly reduced in the control group after 12 months. A caloric restriction was not a primary aim of the Mediterranean diet intervention, except for women with a BMI >35 kg/m^2^, but was obviously anticipated in the control group receiving only one nutrition course. The median proportion of dietary fat of TEI was 40% in both groups and therefore above the recommendation value of 30% [39]. Participants of the NVS II had a median fat intake of 35%.

According to Léon-Munoz et al. [41] a MEDAS score ≥ 9 shows strong adherence, and a MEDAS score ≥ 7 modest adherence to the Mediterranean Diet (MD). As expected, there was almost no conformance in our study population in both groups to a MD pattern before the intervention [41]. The median baseline score of our study cohort of 7 (range 2–10) in the IG and 5 [3–11] in the control (*p* = 0.020) is comparable to the results of the Spanish cohort of the general population (mean ± SE: 6.34 ± 0.03) [41], but lower than the baseline values reported in the PREDIMED study [14] (mean ± SE: Mediterranean Diet with extra virgin olive oil: 8.95 ± 1.79; MD with Nuts: 8.92 ± 1.92; Control diet: 8.42 ± 1.81). So far, there is no data on MEDAS scores in the general German population.

As MEDAS was not a stratification parameter, unfortunately a random group difference was observed at baseline, which persisted during study timeline. However, during the 12-month nutrition education, the intervention group increased their MEDAS value compared to baseline. According to Léon-Munoz et al., these participants with a median MEDAS score of eight obviously present a better adherence to the MD after intervention than the control with six score points [41], despite this consistent group difference.

The participants of the control group, which at baseline received a single group lesson on general information about healthy nutrition according to recommendations of the German Nutrition Society (DGE), expectably did not achieve much higher MEDAS results; however, a slight increase in MEDAS appears at V1 and remains at V2. The participants might have been motivated to fulfill recommendations of the DGE, and there is a considerable overlap between DGE recommendations on healthy nutrition and the typical MD pattern. Therefore, the contents of the group sessions on MD seem to be successfully implemented especially by the intervention group.

Data on physical activity and improvement of physical fitness during intervention are equivocal. On the one side physical activity assessed per questionnaire decreased during active intervention over the first 3 months and unexpectedly increased in the usual care group (Table 5). In contrast, aerobic cardiopulmonary fitness improved in the intervention group, a finding directly related to increased regular physical activity. Physiologically this cannot readily be explained and it seems likely that either the questionnaire assessments were inaccurate, or the participants in the intervention group exercised deliberately for supervised training sessions in the center, thereby improving physical fitness, but did not increase, but rather decreased daily physical activity.

The health related quality of life measured in our trial by EORTC-QLQ-C30 was in the reference range and showed neither differences between the arms nor during intervention. The baseline stress levels in the study population were within the reference range of the German population (age 31–59 years). However, after 12 months stress was significantly reduced probably due to the active lifestyle intervention, illustrated by significantly lower stress scores compared to the control group. These results confirm previous results that lifestyle intervention has a positive influence on mental health [15–17, 42, 43].

## Conclusions

In conclusion, the results of this pilot study were positive regarding feasibility outcome measures such as completion of the study, adherence, safety and satisfaction with the study. Additionally, the intervention indicated that relevant beneficial effects on body weight, stress levels and changes in nutritional behavior towards a Mediterranean-eating pattern can be achieved. Data are, however, limited to a small group of patients. These results will form the basis for a larger randomized trial (LIBRE-2) with an estimated sample size of 600 *BRCA1* and *BRCA2* mutation carriers aiming at improvement of BMI, aerobic and maximal exercise capacity and adherence to a Mediterranean diet [44]. This large intervention study will generate first data on whether breast cancer incidence and prognosis can be influenced by lifestyle intervention. Results of such a large multicenter intervention trial would have a significant impact on clinical recommendations and guidelines for breast cancer prevention.

## Abbreviations

BMI: Body mass index
CG: Control group
DGE: German Nutrition Society (Deutsche Gesellschaft Ernährung)
EFSA: European Food Safety Authority
GC-HBOC: German Consortium for Hereditary Breast and Ovarian Cancer
IG: Intervention group
LIBRE-1: Lifestyle Intervention study in women with hereditary breast and ovarian cancer, 1 = pilot
MD: Mediterranean diet
NVS II: German National Nutrition Survey II
TEI: Total daily calorie intake
